# MIP-C: A new autoimmune rheumatic disease concomitant with the COVID-19 pandemic

**DOI:** 10.2478/rir-2024-0018

**Published:** 2024-10-21

**Authors:** Katja Brion, Mia Phillips, Antonio La Cava

**Affiliations:** Department of Medicine, University of California Los Angeles, Los Angeles, CA, USA; Department of Environmental Science, Management, and Policy, University of California Berkeley, Berkeley, CA, USA; Department of Medicina Molecolare e Biotecnologie Mediche, Federico II University of Naples, Naples Italy

A distinct subtype of dermatomyositis (DM) characterized by high incidence of interstitial lung disease (ILD), skin ulceration and the absence of muscle weakness has been associated with circulating antibodies against melanoma differentiation-associated protein 5 (MDA5).^[1]^ In this subtype of DM with autoantibodies to MDA5, ILD is present in 50%–100% cases and significantly contributes to elevated disease mortality.^[1]^

The etiology of anti-MDA5-positive DM remains elusive, despite epidemiological studies have pointed to a possible viral etiology, supporting the known activity of MDA5 as a viral RNA sensor.^[2]^ Interestingly, during the corona virus disease 2019 (COVID-19) pandemic, case reports and case series have described an emergence of anti-MDA5 antibody-positive myositis and ILD.^[3]^

## A New Autoimmune Rheumatic Disease Associated with the COVID-19 Pandemic

RNA viruses and other pathogens have long been posited as triggers for DM.^[4]^ Taking advantage of the COVID-19 pandemic as an opportunity to study the effect of an RNA virus on the incidence and phenotypes of DM, Costin *et al*. reported an increase in the incidence of juvenile DM in 2020–2021.^[5]^ More recently, David and coworkers investigated the increased incidence of anti-MDA positivity in Yorkshire (United Kingdom) during the COVID-19 pandemic in relation to DM.^[6]^

**Figure 1:**
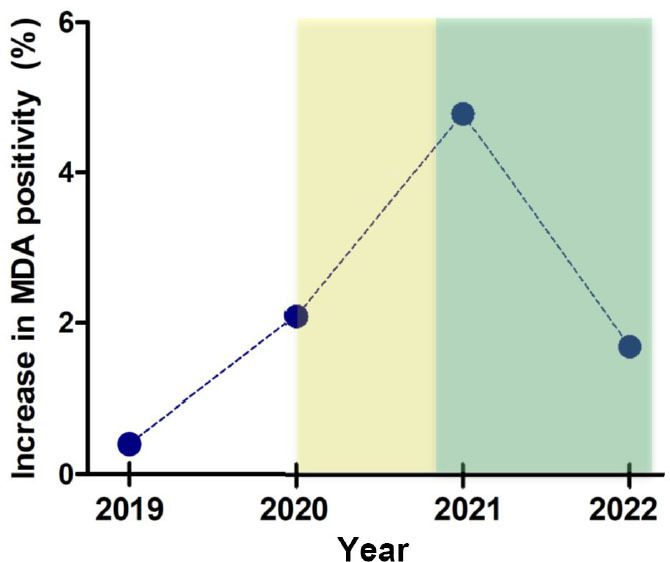
Rates of new anti-MDA5 positive subjects identified by David et al.^[6]^ in their cohort. The yellow shared area encompasses the time of COVID-19 from insurgence as indicated by the first confirmed cases in the UK, the green area represents the period of COVID-19 vaccination campaign in the UK. MDA, melanoma differentiation-associated protein.

Leveraging on the availability of clinical data of subjects with anti-MDA5 antibodies that included information on COVID-19 infection and/or vaccination to severe acute respiratory syndrome coronavirus 2 (SARS-CoV-2), the authors performed a retrospective observational study investigating the association between COVID-19 and anti-MDA5-positive DM. They noted that the rates of new anti-MDA5 positive subjects that in the region had been 1.2% in 2018 and 0.4% in 2019, raised during the COVID-19 pandemic to 2.2% in 2020 and 4.8% in 2021 and started to decline concomitantly with the campaign of COVID-19 vaccination (Figure 1). Numerically, the new cases of anti-MDA5 positivity increased from a total of 6 in the years 2018–2019 to 60 in 2020–2022.^[6]^ In the newly diagnosed anti-MDA5-positive DM individuals, Caucasians had a higher prevalence of the disease (53.3%) as compared to East Asians (5%), although anti-MDA5 positive DM typically affects East Asians disproportionately.^[1]^ Interestingly, there were clinical differences between canonical anti-MDA5 positive DM and anti-MDA5 positive DM patients, leading the authors to coin the term “MDA5 autoimmunity and interstitial pneumonitis contemporaneous with the COVID-19 pandemic”(MIP-C) for this new disease. Compared to classical anti-MDA5 positive DM, MIP-C had a lower incidence of ILD (41%) but more common proximal myopathy.^[7]^ The authors did not address whether a potential reason for the lower incidence of ILD could have been the ethnic makeup of the patients studied – a possibly relevant aspect considering that the rates of ILD in Caucasian patients are lower (38%–73%) than those in Asian patients (82%–100%).^[8]^ The lower mortality rate of MIP-C as compared to anti-MDA5 positive DM might also been possibly associates with demographics, although this was not investigated.

It had previously been reported that seropositivity to MDA5 in COVID-19 patients correlated with disease severity and unfavorable outcomes.^[9]^ David *et al*. found a strong overlap between the surge in anti-MDA5 positive DM and the COVID-19 pandemic (Figure 1). This correlation between MIP-C and SARS-CoV-2 infection was supported by the finding that the majority of the anti-MDA5-positive DM cases had either COVID-19 or, later, vaccination prior to development of DM.

The authors then screened databases and datasets with immunophenotypes and gene signatures in bronchoalveolar lavage fluid of COVID-19 lungs, to add mechanistic information on the reported rise of anti-MDA5 positive DM during the COVID-19 pandemic. They found induction of the gene encoding MDA5 protein, interferon induced with helicase C domain 1 (*IFIH1*)together with that of the interleukin-15 (IL-15)-centric type 1 interferon (IFN) response and alveolar type 2 (AT2) cytopathy. 2D cultures of lung cells and exosome vesicles from COVID-19 patients confirmed *IFIH1* and gene signatures of AT2 cytopathies and autoimmune ILD, linking the exposure to SARS-CoV-2 to *IFIH1* induction. Subsequent studies on peripheral blood mononuclear cells (PBMCs) from acute and convalescent COVID-19 subjects showed that the induction of *IFIH1* positively correlated with increased type 1 IFN activity, IFN stimulated gene 15 positive (ISG15^+^) CD8^+^ cytotoxic T cell signature, and IFN response, *i*.*e*. all factors known to increase the risk for ILD in anti-MDA5-positive DM. Together with the finding that the treatment with systemic corticosteroids decreased IFIH1 expression in subjects carrying selected *IFIH1* variants, these results suggest an interplay between genotype, age, gender and pharmacologic treatment that converges on the levels of expression of IFIH1 during the inflammatory response to SARS-CoV-2. Incidentally, the single nucleotide polymorphism (SNP) rs1990760 of *IFIH1* was the strongest predictor of *IFIH1* induction, while the rs1990760 TT variant conferred protection by suppressing induction of *IFIH1*, IFN, and ISG15^+^ CD8^+^ T cell signatures.

## Conclusions

This work reveals the emergence of a new autoimmune rheumatic disease during the COVID-19 pandemic. This disease is characterized by a distinct clinical presentation from classical anti-MDA5-positive DM, as confirmed by transcriptomic studies. Unanswered questions remain on the etiopathogenesis of MIP-C. For example, an investigation on the interaction of MDA5 with the SARS-CoV-2 Nsp8 protein could provide interesting answers. Since the interaction of Nsp8 with MDA5 and tripartite motif containing 4 (TRIM4) shields MDA5 from polyubiquitination,^[10]^ the evasion from recognition of the viral RNA might allow SARS-CoV-2 to favor anti-MDA5 autoimmunity, although this needs to be addressed. Other aspects worth of future investigation could be whether SARS-CoV-2 can trigger autoimmunity to MDA5 through molecular mimicry, epitope spreading, bystander activation, *etc*., considering that the link between COVID-19 and MDA5 autoimmunity became nuanced when comparing rates of MDA5 positivity to average daily COVID-19 cases. In sum, although the observational nature of the study by David *et al*. leaves several mechanistic questions open, including how to extrapolate the findings to other ethnic populations from different geographic areas, the link between anti-MDA5 autoimmunity and COVID-19 in MIP-C leads to appreciate and wonder about yet unknown health consequences of the burden caused by the COVID-19 pandemic.
